# Advancing Real-Time Food Inspection: An Improved YOLOv10-Based Lightweight Algorithm for Detecting Tilapia Fillet Residues

**DOI:** 10.3390/foods14101772

**Published:** 2025-05-16

**Authors:** Zihao Su, Shuqi Tang, Nan Zhong

**Affiliations:** 1 College of Engineering, South China Agricultural University, Guangzhou 510642, China; suzihao@stu.scau.edu.cn (Z.S.); sqtang@stu.scau.edu.cn (S.T.); 2 Guangdong Provincial Key Laboratory of Agricultural Artificial Intelligence, Guangzhou 510642, China

**Keywords:** lightweight model, YOLOv10, object detection, tilapia processing

## Abstract

Tilapia fillet is an aquatic product of great economic value. Detection of impurities on tilapia fillet surfaces is typically performed manually or with specialized optical equipment. These residues negatively impact both the processing quality and the economic value of the product. To solve this problem, this study proposes a tilapia fillet residues detection model, the double-headed GC-YOLOv10n; the model is further lightweighted and achieves improved detection performance compared to the double-headed GC-YOLOv10n. The model demonstrates the best overall performance among many mainstream detection algorithms with a small model size (3.3 MB), a high frame rate (77FPS), and an excellent *mAP* (0.942). It is able to complete the task of tilapia fillet residues detection with low cost, high efficiency, and high accuracy, thus effectively improving the product quality and production efficiency of tilapia fillets.

## 1. Introduction

Tilapia is one of the important high-quality species promoted by the Food and Agriculture Organization of the United Nations (FAO) for global aquaculture, which is popular because it is rich in protein, vitamins, minerals, and multiple unsaturated fatty acids [1]. Tilapia is one of the most economically valuable freshwater fish in China’s aquaculture industry with strong domestic and international market demand, particularly for processed products such as frozen fillets [2,3]. The processing of tilapia fillets involves deheading, scaling, gutting, and cleaning [4]. In China, over 80% of primary fish processing is performed manually, which can affect the product quality as fillets often retain scales, bones, and other impurities. Currently, to reduce residues in the fillets, the processing typically employs a multi-pass cleaning method. However, this approach is inefficient, may not completely remove residues, and could even lead to the deterioration in fillet quality due to excessive manipulation. To address this problem, there is an urgent need for a low-cost, high-efficiency, high-accuracy, and non-destructive method for detecting tilapia fillet residues.

With the development of computer vision technology and optical equipment, machine vision has been increasingly used in aquatic product quality inspection. However, in the field of fish fillet residues detection, traditional techniques still face many challenges. Detection approaches based on traditional machine learning [5] are often limited by manually designed features, leading to poor representation, lower detection accuracy, and weak generalization. In order to obtain richer features, researchers combine special optical imaging techniques, such as hyperspectral imaging [6,7,8], X-ray imaging [9,10,11], and ultraviolet fluorescence imaging [12], to enhance the detection of fish fillet residues. However, these methods usually rely on expensive hardware equipment, and although they exhibit high detection accuracy in laboratory settings, their high deployment cost limits their popularity in processing plants. In addition, most of these traditional methods are designed to detect a single target, making it difficult to deal with multiple residues in fish fillets at the same time, and thus have obvious shortcomings in improving detection efficiency and adapting to diverse tasks.

With the rapid development of deep learning technology, object detection algorithms provide new solutions for fish fillet residues detection. Deep learning methods can achieve high accuracy and robustness in multi-target detection tasks by training neural networks without relying on complex hardware. Currently, object detection has been widely used in the field of food detection, such as the detection of Atlantic salmon fish bone, tomato, corn, etc., based on Faster-RCNN [13,14,15], the detection of corn kernels, Atlantic salmon fillet residues, foreign objects in chili peppers, pears, etc., based on the YOLO series of algorithms [16,17,18,19], and the detection of green plums, strawberries, etc., based on transformers [20,21]. However, mainstream object detection models (e.g., Faster R-CNN, YOLO series, etc.) still face two major challenges when applied to fish fillet residues detection [14,17]: firstly, the number of parameters and computational volume of the model are large, which makes it difficult to achieve efficient deployment on low-computing power devices; secondly, the detection of fish fillet residues is characterized by small target detection, for which the detection performance of existing models needs to be improved. To address the above problems, this paper proposes a lightweight algorithm that can detect blood clots, bones, viscera, and scales on tilapia fillets in real time, and features high accuracy and a low deployment cost. YOLOv10n was selected as the benchmark model in this paper due to the high real-time performance of the YOLO series. Compared to transformer-based detection models, YOLOv10n is more lightweight and better suited for deployment on mobile or resource-constrained devices. Additionally, it tends to perform more robustly on small-scale datasets, making it a suitable choice for the limited data scenario in our task. The main contributions of this paper are as follows: (1) A tilapia fillet residues dataset containing multiple fish tissue residues was created. (2) A double-headed GC-YOLOv10n model is proposed, which significantly reduces the number of model parameters and computational effort while obtaining improved detection results. (3) The effectiveness and applicability of the dual-head GC lightweight method were verified. Through comparative experiments, it was confirmed that the double-headed GC-YOLOv10n has the best comprehensive performance in the detection of fish fillet residues.

## 2. Materials and Methods

### 2.1. Dataset Acquisition and Construction

Fresh tilapia were purchased at the Yunlu Market in Foshan, Guangdong, China. In order to design our experimental samples collection process based on the real-world production environment and typical tilapia fillet processing workflow, the fish were prepared at the market, including descaling and head removal. To simulate manual gutting on a production line, the researchers manually disemboweled and gutted in a kitchen environment to replicate the real process as closely as possible.

In the experiment, a Xiaomi 13 smartphone was used as the image acquisition device, which has a high resolution and good imaging quality. In order to ensure the consistency of the shooting angle and the stability of the lighting conditions, the phone was fixed in the center of the ring LED fill light. The samples were photographed at a vertical downward angle, and the distance between the phone and the samples was about 20 cm. The rated power of the ring LED fill light was 105 W, with three levels of brightness adjustment, and the middle level was selected to provide high luminous flux and a uniform lighting effect.

The image acquisition parameters were set to ISO 400 to ensure that the images with appropriate brightness and low noise were obtained under the supplementary light conditions. The final resolution of the acquired image was 4096 × 3072, which meets the requirements of subsequent target detection and image processing tasks on image details.

Mosaic [22] data augmentation was performed on the obtained raw images. A mosaic data enhancement algorithm is used to stitch four random images to form a new image, which achieves an increase in the diversity of the data, and at the same time, since the new image mixes the semantic information of the four original images, it allows the model to detect targets beyond the regular context, which greatly enhances the model’s generalization ability.

**Figure 1:**
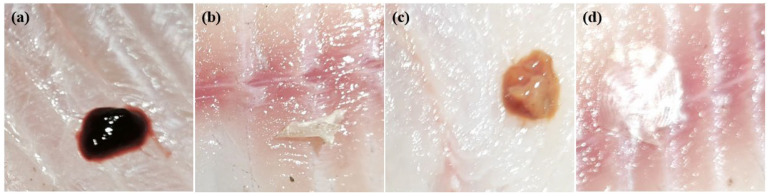
Different types of tilapia fillet residues: (**a**) blood, (**b**) bone, (**c**) viscera, (**d**) scale.

According to the practical knowledge of tilapia fillet processing in actual production lines, the residues of processed tilapia fillets were categorized into four types: blood, bone, viscera, and scale in this paper, and their images are shown in Figure 1. A total of 481 valid image samples were obtained in the experiment, of which 451 positive samples contained one or more types of residues, and a small number of negative samples, i.e., a total of 30 tilapia fillets without any residues, were set up in order to reduce the false detection rate and improve the model robustness.

The image samples were manually annotated using the LabelImg tool, with labels initially saved in the Pascal VOC format. A Python script was then used to convert the annotations into YOLO format, generating the corresponding .txt files. Following the directory structure required by the YOLOv10 project, the dataset was split into a training set with 421 images and a validation set with 60 images. During the splitting process, we ensured that the four residue categories (blood, bone, viscera, and scale) were evenly distributed across the two subsets to maintain class balance. This finalized dataset was used throughout the experiments.

### 2.2. Training

Featurize is a cloud computing power leasing platform, which provides a fast and convenient deep learning algorithm development environment. In order to train efficiently and stably, Featurize was chosen to train the model we proposed in the article, and the selected cloud server configuration is shown in Table 1.

In actual model training, most models stop early at 300–500 epochs because the model no longer shows significant improvement in detection on the validation set, so in order to enable the model to fully learn residues features, the iteration number of this experiment is set to 500 epochs. To optimize the GPU computational power of the cloud server, the batch size is set to 32.

To mitigate the effects of randomness during training, all experiments in this paper are conducted using three different random seeds (0, 1, and 2). We report the mean of the evaluation metrics across these runs to ensure a more robust and reliable comparison. This setup improves the reproducibility and fairness of the experimental results.

### 2.3. Theoretical

#### 2.3.1. YOLOv10

In the past few years, for the YOLO family of algorithms, as an excellent object detection algorithm, the continuous improvement in the model architecture during its development has played an important role in improving the detection speed and accuracy of the object detection task. YOLOv10 [22] is the most recent version of the YOLO family of algorithms, and the most significant difference and innovation of YOLOv10, relative to the other YOLO family algorithms, is that it no longer relies on non-maximum suppression (NMS) post-processing during the target’s prediction phase, which greatly improves the model performance and reduces the inference latency, enabling better end-to-end deployment of the model. To achieve NMS-free, YOLOv10 uses a dual-label assignment strategy, where two detection heads are used simultaneously in the training phase of the model, one using one-to-one assignment and one using one-to-many assignment. This approach of using both one-to-many and one-to-one detector heads in the training phase and only one-to-one detector heads in the prediction phase not only enables the model to obtain rich supervisory signals to ensure the model’s performance, but also frees the model from the computational cost of NMS post-processing in actual inference.

In addition, YOLOv10 optimizes some modules, as well as uses some new modules, aimed at reducing the computational overhead while increasing the performance of the model.

SCDown is a lightweight convolution operation and it is used in two places on the backbone for feature downsampling. Unlike ordinary Conv components, SCDown decouples the spatial and channel features of the feature map, and first uses a convolution kernel of size 1 × 1 to perform point-by-point convolution to change the number of channels, and then uses a 3 × 3 convolution kernel to perform deep convolution to realize spatial downsampling, so that spatial channel decoupling is achieved. This spatial channel decoupling process reduces the computational cost while maximizing information retention. YOLOv10 uses an efficient local self-attention module PSA (Pyramid Squeeze Attention) in the last layer of the backbone. PSA forms a pyramidal feature map with multiple scales by concatenating features convolved with different-sized convolutional kernels, and then applies the SE (Squeeze-and-Excitation Networks) attention mechanism to the feature map to extract a richer feature map. To extract richer feature information, YOLOv10 also uses the C2fCIB module at the deepest level of the neck position, which improves on the C2f [23] by replacing the Bottleneck in the C2f using the CIB [22] module, and since the CIB module is replacing the standard convolution in the Bottleneck with a depth convolution and a point-by-point convolution, this makes the number of parameters in the C2fCIB reduced compared to the C2f, making the model more lightweight.

**Figure 2:**
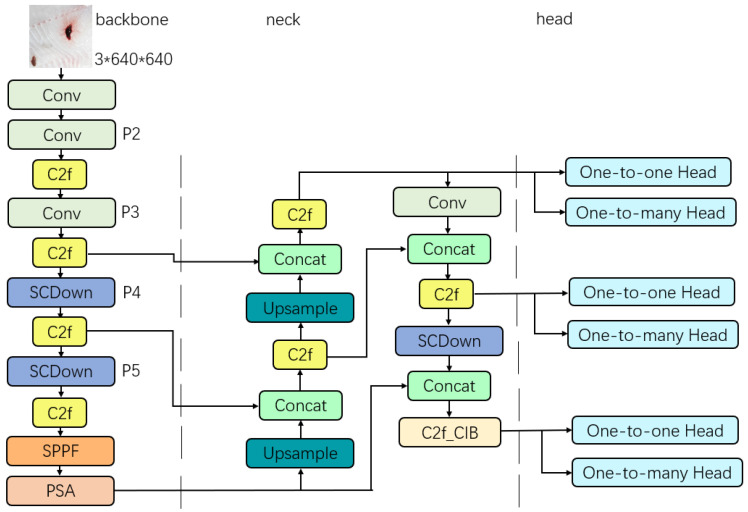
Model structure of YOLOv10n.

YOLOv10 is similar to the previous generations of YOLO algorithms, and is divided into five models according to the size of the model, namely YOLOv10n, YOLOv10s, YOLOv10m, YOLOv10l, and YOLOv10x. The network structure of the different YOLOv10 models is basically the same, with the parameter “Depth” determining the vertical depth of the model, i.e., the number of repetitions of the YOLO components in the model. The number of channels output when the feature map passes through a certain component is determined by the parameter “Width”. These two parameters determine the size of the model, e.g., the depth and width of YOLOv10n are 0.33 and 0.25, respectively, which is the most lightweight and fastest model in YOLOv10, whereas the depth parameter and width parameter of YOLOv10x are 1.00 and 1.25, respectively, which is the largest and relatively slowest one of the models. Generally speaking, in the same series of models, the smaller the model, the faster the speed, the less computation and parameters, but the reasoning ability is also reduced compared with the larger model. Therefore, the choice of model should depend on the focus of the research problem. In this paper, we hope to make the model as lightweight as possible, so that it can be easily deployed on mobile devices under resource-constrained conditions, YOLOv10n consists of a backbone, a neck, and a decoupled head, with approximately 2.7 million parameters. Due to its suitability for deployment on lightweight devices, it was selected as the benchmark model for the research and experiments (Figure 2).

#### 2.3.2. Ghost_Bottleneck Lightweight Module

**Figure 3:**
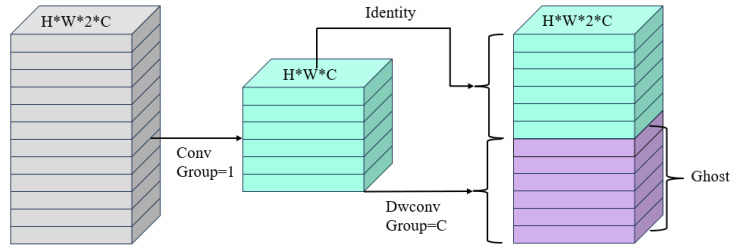
Ghost_module structure diagram.

Ghosh_module proposed by Huawei Noah’s Ark Lab [24] is a lightweight convolution operation, and its core idea is to decompose ordinary convolution into two steps; the first step is to halve the channels of the input feature maps through a 1 × 1 convolution kernel in order to reduce the amount of the subsequent computation, while the second step is to perform feature map-by-feature map, i.e., deep convolution on the obtained feature maps, and finally, the feature maps obtained by two steps are stitched together to realize feature fusion. In this way, Ghost_module can obtain the feature maps with the same number of channels as the normal convolution with less overhead. The output feature maps after normal convolution will have many similar features, which will cause redundancy of information, so even though Ghost_module has fewer parameters and less computation compared to normal convolution, it will not decrease the learning effect of the network on the features too much. Figure 3 shows the structure of Ghost_module.

Bottleneck is an important component in ResNet [25], which is widely used in deep neural networks for feature extraction because it can realize the direct transfer of information between different layers in deep neural networks. This concept of fusion refers to the process of efficiently combining and transmitting feature representations across layers to enhance the model’s capacity to capture complex patterns. By introducing the Ghost_module lightweight convolution operation into Bottleneck instead of the original ordinary convolution operation, we can obtain the Ghost_Bottleneck module, which enables efficient fusion of information from different layers of the network, but with smaller parameters and lower computational complexity.

#### 2.3.3. CIB Module

**Figure 4:**
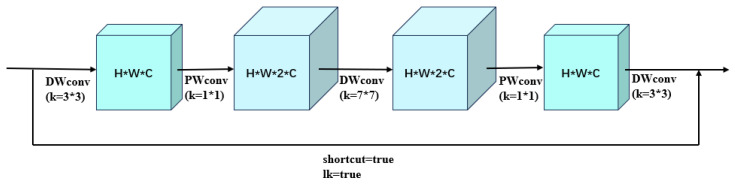
CIB structure diagram.

The authors of the MobileNetV2 paper [25] found that 1 × 1 convolution of the feature map in the network and then using deep convolution to extract the features can make the network model more informative, and since the convolution kernel of deep convolution only convolves one channel of the feature layer, the computation will not be too high even if the features are upgraded and then downgraded to extract the features. This network structure of dimension up and then dimension down is the opposite of the residual structure in the ResNet [26] paper, so it is called an inverted residual structure. Based on this structure, the YOLOv10 [22] paper proposes an inverted residual block CIB, which uses depthwise convolutions to fuse spatial features of the feature map, and at the same time uses 1 × 1 pointwise convolutions to fuse the channel features of the feature map. There are often redundant features piled up at the deep high-dimensional features of the network, and using the CIB module at these locations can increase the efficiency of the model while maintaining or even improving the robustness of the model. Figure 4 shows the structure of a typical CIB module.

#### 2.3.4. YOLO Detection Head

The most common definition of target size comes from the common dataset for object detection, COCO [27], which defines targets smaller than 32 × 32 pixel dots, larger than 32 × 32 smaller than 96 × 96 pixel dots, and larger than 96 × 96 pixel dots as small-scale targets, mesoscale targets, and large-scale targets, respectively.

For the YOLO series of model algorithms, the detection head of its original structure is generally divided into three detection heads, P3, P4, and P5, along with the depth of the network, which represent the model’s detection of small-scale targets, medium-scale targets, and large-scale targets from the top to the bottom. The YOLO model makes use of the feature pyramid network (FPN) [28] for the feature extraction of targets at different scales in the image, which is a bottom-up and then top-down network. An FPN is a bottom-up and then top-down network structure, which achieves feature enhancement by fusing the representational information extracted from the shallow layer of the model with the semantic information extracted from the deeper layer of the network, which enables the model to better obtain the feature information of the small-scale targets, rather than gradually losing the information of the small-scale targets as the depth of the network increases.

For different object detection tasks, the YOLO model can be changed by adding or removing detection heads to make its network structure more suitable for feature learning for targets of specific sizes, thus enhancing the model’s detection performance.

#### 2.3.5. Double-Headed GC-YOLOv10n

In this paper, YOLOv10n is used as a benchmark model, which is lightweighted in order to obtain a model that can be more easily deployed on resource-constrained mobile portable devices. The improvement goal is to reduce the number of parameters and the computational complexity of the model to obtain a smaller and faster model, and at the same time, to make the model more suitable for the tilapia fillet residues detection task, the performance effect should be better than the benchmark model.

Firstly, use the Ghost_Bottleneck lightweight module mentioned above to replace the normal Bottleneck module in the C2f module in YOLOv10n’s backbone, so that the lightweight and efficient convolution of the Ghost_module can be utilized at the model backbone to learn features, reducing a large number of parameters and computation complexity without losing too many effective features.

Secondly, given that the target of this paper is the various fish tissues residues that may remain on tilapia fillets after primary processing, mainly blood, bone, scale, and viscera, it is worth noting that the vast majority of these fish tissue residues are smaller than 96 × 96 pixel points in the self-constructed dataset, so the vast majority of the targets detected in this study are small-scale targets and mesoscale targets. So, the P5 detector head used for detecting large-scale targets in YOLOv10n was removed, and the removal of the large-scale target detector head on the one hand realizes the lightness of the model, and on the other hand reduces the impact of redundant large-scale features on the detection effect.

Thirdly, referring to YOLOv10’s idea of using the C2fCIB module to replace the C2f module at the deeper part of the network, the C2f used in the P4 detection head at the neck is replaced by the C2fCIB.

**Figure 5:**
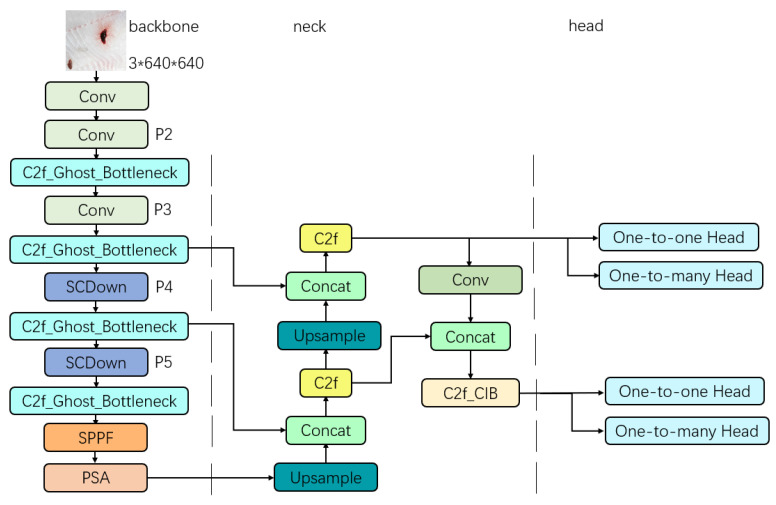
Model structure of the double-headed GC-YOLOv10n.

The above three modifications are collectively referred to as dual-head GC lightweighting, and the double-headed GC-YOLOv10n is obtained by dual-head GC lightweighting of the YOLOv10n, whose model structure is shown in Figure 5. In order to verify the impact of different modifications on the inference performance of the model, this paper conducted ablation experiments.

In the ablation experiments presented in Section 3.1, three modifications of the model were designed to evaluate the contribution of individual components, denoted as A, B, and C, respectively, as follows:

A: Deletion of the P5 large-scale target detection header in the model, B: the use of Ghost_Bottleneck to replace the Bottleneck of the C2f for all C2fs at the benchmark model backbone, C: the replacement of the C2f by the C2fCIB in the P4 detection head at the neck.

To further verify the effectiveness of the proposed dual-head GC lightweighting approach, we extended its application to models with architectural similarity to YOLOv10n. Moreover, to evaluate the overall performance of the double-headed GC-YOLOv10n, comparative experiments were conducted against mainstream object detection algorithms.

### 2.4. Performance Evaluation Metrics

In this paper, the Average Precision (*AP*) of detecting a single target, the mean Average Precision (*mAP*) of multiple targets, the number of model parameters, the model storage size *model_size*, the model’s billionth floating-point computations *GFLOPs*, and the model’s actual detection speed *FPS* were used as the main indexes for evaluating the model.

The *AP* is a metric for a single detected object, which integrates the detection precision and recall of a single detected object, while the object detection task often contains multiple targets to be detected, and the *mAP* obtained by averaging the *AP* of multiple targets is the most widely used metric in the field of object detection. The commonly used *mAP*50 refers to the mean Average Precision (*mAP*) calculated at an Intersection over Union (*IoU*) threshold of 0.5. Meanwhile, *mAP*50-95 refers to the mean of the *mAP* values calculated across multiple *IoU* thresholds ranging from 0.5 to 0.95 with a step size of 0.05, providing a more comprehensive evaluation of the model performance across different levels of overlap between predicted and ground truth bounding boxes.

In general, the detection precision (*P*) and recall (*R*) of a single target exhibit the characteristics of mutual restriction, so the *AP* value of the target is usually obtained by plotting the PR curve. The formulas for *AP*, *mAP*, *P*, and *R* are as follows:

(1) $$AP=\int_{0}^{1}R\left(P\right)dP$$

(2) $$mAP=\frac{1}{n}\sum_{i=1}^{n}AP(i)$$

(3) $$P=\frac{TP}{TP+FP}$$

(4) $$R=\frac{TP}{TP+FN}$$

where *TP* denotes a true positive where both the predicted and true values are positive samples, *FP* is a false positive where the predicted value is a positive sample and the true value is a negative sample, and *FN* is a false negative where the predicted value is a negative sample and the true value is a positive sample. From the formula, it can be seen that the *AP* value of a single target is the area of the PR curve with its recall as the horizontal coordinate and precision as the vertical coordinate.

The number of model parameters is often used as a measure of the spatial complexity of the model computation, which mainly consists of the weight parameters and bias parameters in the model that can be learned through backpropagation, and a larger number of model parameters implies a larger *model_size* of the model storage size. Although larger models can learn the target’s features better and fit more complex features, we should also consider the impact of the model size on overfitting risk, as larger models with more parameters tend to overfit. The relationship between the model size and overfitting was monitored by evaluating the model’s performance on both the training and validation sets. Models with a larger number of parameters were expected to show a greater discrepancy between training and validation performance, suggesting an increased risk of overfitting. And deploying large models on resource-constrained mobile devices is extremely difficult.

Model Billion Floating-Point Operations (*GFLOPs*) are commonly used to measure the time complexity of network model computation, which refers to the number of floating-point operations (in units of 10^9^) that need to be performed during the training or inference process of a model on a target. So, the higher the *GFLOPs*, the more computational resources are required by the model, which is usually reflected in the time required for the model to complete training or inference.

*FPS* refers to the number of images that the model can process per unit of time, which is an important indicator of the suitability of the model for time-sensitive tasks. The larger the *FPS*, the better the real-time detection capability of the model and the faster the inference of the images.

## 3. Results and Discussion

Our work demonstrates advancements in real-time food detection, as various experiments have proven improvements in speed and model size while maintaining excellent detection performance. In terms of computational capability, this lightweight model is essential for deployment in resource-constrained devices.

### 3.1. Experimental Results and Analysis of Ablation with Improved Algorithm

In order to verify the effectiveness of the dual-head GC lightweighting proposed in this article for the YOLOv10n benchmark model in improving the model performance, seven groups of experiments were set up under the premise of ensuring that only the model is changed and all other various hyper-parameters and environment variables are the same. Table 2 demonstrates the experimental results of the seven groups of experiments with the YOLOv10n original model experiments for a total of eight groups of experiments; the meaning of A, B, and C in Table 2 and Table 3 has been explained in Section 2.3.5 of this paper. Ticking a module indicates that the corresponding improvement method is used for the benchmark model. The table of the detection results of the experiments on individual targets is shown in Table 3.

From Table 2, it can be seen that the double-headed GC-YOLOv10n after the dual-head GC lightweighting is compared with the benchmark model YOLOv10n, and the parameters, *GFLOPs*, and *model_size* decrease to 54.2%, 75%, and 56.9% of the benchmark model, respectively. And the superiority of the double-headed GC-YOLOv10n is that the detection performance of the model is better while both the model space complexity and computational time complexity undergo a significant decrease. Compared to YOLOv10n, the double-headed GC-YOLOv10n was more lightweight while achieving improved performance across most evaluation metrics. Specifically, the mAP50, recall, and precision increased by 1.0%, 1.2%, and 1.2%, respectively. Although the *mAP*50-90 decreased slightly by 0.8%, the reduction in model complexity offered a favorable trade-off between accuracy and efficiency, making the model more suitable for deployment in resource-constrained environments. Moreover, the lighter architecture led to a 21 *FPS* increase in inference speed; this further enhances the model’s ability for real-time detection on mobile devices with limited computational resources.

When analyzing the effects of individual improvements on the benchmark model YOLOv10n in Table 2, we can draw the following conclusions: (1) When only the large-scale target detection head P5 is removed, the number of model parameters significantly decreases to 73.2% of the benchmark model. Since the object of study in this paper is mainly small target objects such as blood clots and scales on tilapia fillets, the model’s effectiveness in detecting small targets is improved to a certain extent with the removal of the large-target detection layer, and the *mAP* value reaches 0.937. This result confirms the validity of the change in the model detection head for the size-specific detection task, and this idea is also discussed in the studies of Wang et al. [29] and Mu et al. [30]. (2) In the experiments where only the C2f at the model backbone is replaced with the C2f_Ghost_Bottleneck, although its streamlining effect on the number of model parameters is not as significant as deleting the P5 detection header, the lightweight convolutional property of the Ghost_module results in a significant decrease in the computational GFLOPs of the network model. This property was also confirmed in the studies of Zhang et al. [31] and Yuan et al. [32] who constructed lightweight models based on Ghost_module. However, since Ghost_module may ignore some effective features while removing redundant features in the network, the detection effect of the model shows some degree of degradation. (3) In the experiment using the C2fCIB module in the P4 detection head at the model neck, we verified the positive effect of the module as stated by Wang et al. [22]. Its *mAP*50-90, recall, and precision of the model showed a certain degree of degradation without basically lightening the benchmark model YOLOv10n, but the most important metric, *mAP*50, was improved by 0.8%.

Subsequent ablation experiments with two sets of improvement points further confirmed the positive effects of each improvement point on the number of model parameters, the size of floating-point computation and the detection effect of the benchmark model, which are summarized as follows: deleting the P5 detection head and using the Ghost_Bottleneck in the C2f of the model backbone both make the network model more lightweight, and at the same time, the former makes the new model more suitable for the small-scale target detection task, while the latter causes a slight decrease in the ability of the model to learn features but still has a good fitting ability. The use of the C2fCIB in the P4 detection head at the model neck significantly enhances the detection performance of the model.

The performance of the double-headed GC-YOLOv10n in detecting fish residues—including bones, blood clots, scales, and viscera—can be observed from Table 3. The *mAP* for fish bone detection is 3% higher than that of the baseline YOLOv10n, indicating a substantial improvement in identifying fish bones. For blood clots, due to their more distinctive color features, both models achieve over a 99% *mAP* with negligible performance difference. In the case of fish scales and viscera, although the *mAP* improvements are relatively small (0.1% and 0.2%, respectively), the recall and precision show noticeable gains: 3.4% and 2.7% for scales, and 1.3% and 3.0% for viscera. These improvements effectively reduce the rates of missed and false detections for these classes. And similar trends were consistently observed across all runs, which supports the robustness and reliability of the conclusions drawn.

**Figure 6:**
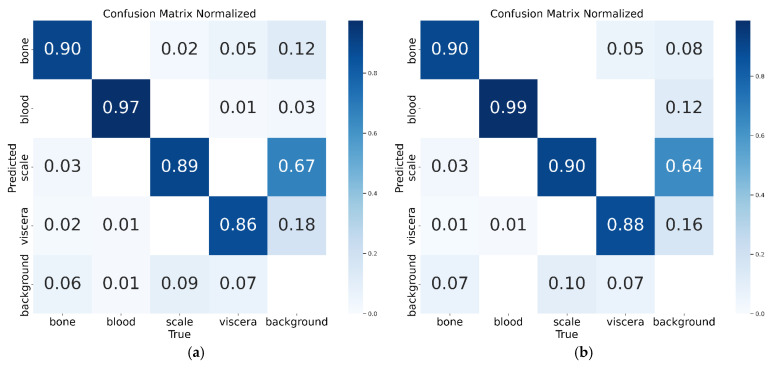
Confusion matrix for (**a**) YOLOv10n; (**b**) double-headed GC-YOLOv10n.

**Figure 7:**
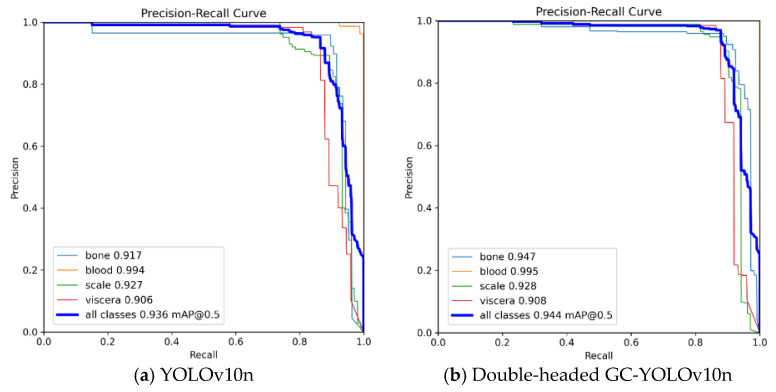
PR plots of (**a**) YOLOv10n; (**b**) double-headed GC-YOLOv10n.

In summary, except for the detection performance of blood clots, which is close to the upper limit of 100% and is difficult to obtain further improvement, compared with the benchmark model YOLOv10n, the detection ability of the double-headed GC-YOLOv10n on fish bones, fish scales, and viscera has been significantly improved. For the convenience of visual performance comparison, Figure 6 and Figure 7 show the confusion matrices and PR curves of YOLOv10n and the double-headed GC-YOLOv10n, respectively. These visualizations are based on the best-performing run among the three random seeds, and serve as representative examples, as similar patterns were observed across all runs.

### 3.2. Validation Experiment on the Applicability of the Dual-Head GC Lightweighting Method

The setup experiments apply the dual-head GC lightweighting method proposed in this paper to different-sized models of YOLOv10 and YOLOv8 [23], which have a similar structure to YOLOv10. The detection results before and after the use of the lightweighting method in different-sized models were compared to verify the effectiveness of the lightweighting method. The experimental result data of applying this lightweighting method to YOLOv10s, YOLOv10l, YOLOv8n, and YOLOv8s are shown in Table 4.

It was easy to see that the lightweight method can reduce the number of parameters of each model to nearly half or even more of the original model, while making the model computation drop and the model inference speed rise. When the benchmark models are larger, such as YOLOv10l and YOLOv8s, the storage size (*model_size*) of their models decreases even more significantly, both exceeding 50%. More importantly, except for the larger YOLOv10l whose *mAP* remains unchanged after being lightweighted, all other models lightweighted by the dual-head GC gain an increase in detection performance, and the lightweighted YOLOv10s, YOLOv8n, and YOLOv8s have an increase in *mAP* of 1.2%, 0.6%, and 0.8%, respectively. The results demonstrated that the proposed dual-head GC lightweighting method was applicable across multiple YOLO object detection algorithms. It effectively reduced the model parameters and computational cost, while enhancing the inference speed, without notable degradation in detection performance. Moreover, by enabling more efficient extraction of small-scale target features at the backbone and improved feature fusion at the neck, the lightweight models even achieved a slight improvement in detection accuracy.

### 3.3. Experimental Results and Analysis of the Comparison Between Double-Headed GC-YOLOv10n and Mainstream Object Detection Algorithms

Comparison experiments were conducted between the double-headed GC-YOLOv10n and current algorithms commonly used in object detection: YOLOv5n, YOLOv5m [33], siwn-transformer [34], rt-detc [35], ShuffleNetV2 [36], and MobileNetV3 [37], and the experimental results of each model are shown in Table 5.

Among the object detection models listed in Table 5, YOLOv5m exhibited the highest *mAP* and the best detection effect. However, its advantage is not obvious, which is only 0.2% higher than that of the double-headed GC-YOLOv10n, and it is worth noting that the model parameters and computation of YOLOv5m are 17 and 10 times higher than those of the double-headed GC-YOLOv10n, respectively, while its detection speed metric, *FPS*, is 42 frame s^−1^ lower, and its real-time detection capability is much less than that of the double-headed GC-YOLOv10n. In comparison with the size of YOLOv5n, ShuffleNetV2, and MobileNetV3, the *mAP* of the double-headed GC-YOLOv10n was 0.8%, 1.8%, and 1.6% higher, respectively. In particular, at a high *IoU* (*mAP*50-90), the detection of the double-headed GC-YOLOv10n was significantly better than that of the commonly used lightweight models ShuffleNetV2 and MobileNetV3 by 4% and 3.1%, respectively. In addition, compared with Swin-Transformer and RT-DERT, which have been popular transformer-based object detection models in recent years, the double-headed GC-YOLOv10n is only 2.9% and 4.5% of theirs in terms of the model parameters, and 4.7% and 5.8% of theirs in terms of the model computation, although the model lightweighting is so high that the double-headed GC-YOLOv10n still outperforms them by 0.9% and 5.5%, respectively, in the detection effect index *mAP*50. This may be because transformer-based models are better suited for detection tasks that involve large datasets, while the double-headed GC-YOLOv10n can achieve better detection performance with a lightweight network when the amount of data are smaller.

**Figure 8:**
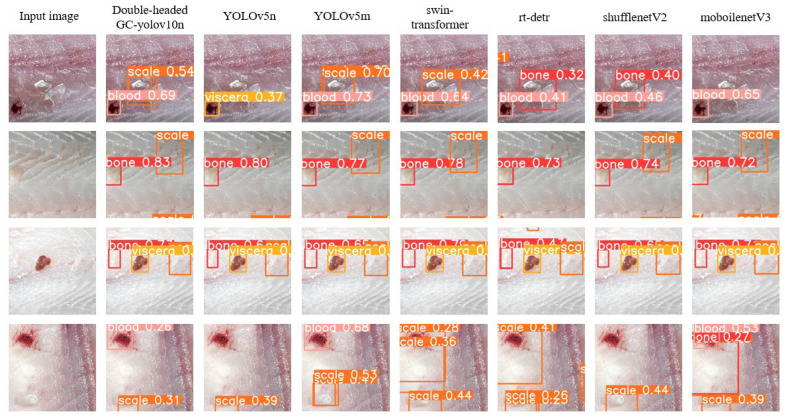
Detection maps for different object detection algorithms.

In order to compare more intuitively the effectiveness of different algorithms in detecting tilapia residues, we carefully selected four images containing different types of residues and applied the above algorithms to detect them, respectively. The detection results are shown in Figure 8; the pixel size of each image is 400 × 400. Among them, the two algorithms, the double-headed GC-YOLOv10n and YOLOv5m, show excellent detection capabilities, and they accurately identify all the residues in the images, which matches the conclusions of Table 5, and further confirms the superiority of the two algorithms with high recall. In contrast, RT-DERT has a relatively poor detection effect, with omissions and misdetections of fish scales, while Swin-Transformer has an excellent overall detection effect, but has the same misdetection of background as fish scales as RT-DERT. In addition, the YOLOv5n, ShuffleNetV2, and MobileNetV3 algorithms, which are similar in scale to the double-headed GC-YOLOv10n, also show a certain degree of leakage and misdetection during the detection process, and their overall detection performance is obviously not as good as that of the double-headed GC-YOLOv10n.

In summary, in the comparison experiments for the current popular object detection models used in this dataset, the double-headed GC-YOLOv10n achieves the best balance between model lightweighting and detection effectiveness. With a model storage size of only 3.3 MB, it achieves an *mAP* of 94.2% as well as an *FPS* of 77. Thus, the double-headed GC-YOLOv10n can be well applied to real-time demanding and resource-constrained tasks with very high detection accuracy. Although the double-headed GC-YOLOv10n has shown good results, we also acknowledge several limitations of our current study. Firstly, the sample size of our dataset is relatively small, which may constrain the model’s generalization to more diverse real-world scenarios. Specifically, the model occasionally misidentifies fish scales as background, particularly when their texture and color closely resemble those of the surrounding area. This issue is likely exacerbated by the limited variability in the dataset. In addition, while we employed three different random seeds to mitigate training randomness and improve reproducibility, this may still be insufficient to support strong statistical significance. A larger number of independent runs would be needed for more rigorous significance testing and confidence in the observed performance trends.

To address these limitations, future work will focus on expanding the dataset with a greater variety of samples, including different fish species, environmental conditions, and lighting scenarios, as well as performing more extensive experiments to enhance the reliability and statistical robustness of the evaluation.

## 4. Conclusions

In this paper, a lightweight double-headed GC-YOLOv10n is proposed using YOLOv10n as a benchmark model, and the model is used in a self-constructed tilapia fillet dataset for tilapia fillet residues detection. Considering that the objects studied in this paper are mostly small-scale targets, the P5 large-scale target detection head is firstly deleted, and in order to further realize the lightweighting of the model, the Ghost_module lightweight convolution module is used to transform the C2f module at the backbone, and then the CIB module is used for the feature map that fuses the two different-scale target features at the neck in order to further realize the redundant feature removal. The final double-headed GC-YOLOv10n is reduced to 54.2%, 75%, and 56.9% of the benchmark model in terms of the number of model parameters, the amount of model float computation, and the size of the model, respectively, and on the basis of this lightweighting, the *mAP*, recall, and precision still gain 1%, 1.2%, and 1.2%, respectively. Although the *mAP*50-90 slightly decreases by 0.8%, the overall trade-off between accuracy and efficiency remains favorable. The effectiveness of the dual-head GC lightweighting method is further verified by subsequently applying the three improvements to YOLOv10’s models of different sizes, YOLOv10s and YOLOv10l, as well as YOLOv8n and YOLOv8s, which are similar in structure to YOLOv10. Finally, by experimenting and comparing the detection effect of the double-headed GC-YOLOv10n with several popular object detection algorithms on a self-constructed tilapia fillet dataset, it is confirmed that the double-headed GC-YOLOv10n can strike a good balance between the lightweighting effect and the detection performance, and is suitable to be deployed on resource-constrained mobile devices to detect tilapia fillet residues in real time and accurately. It should be noted that this work is preliminary, as it is based on a small-scale dataset, and the replication experiments were only conducted by running the algorithms multiple times with different random seed settings.

## Figures and Tables

**Table 1:** Computer configuration.

Configure	Parameters
GPU	RTX 4090
CPU	16-core AMD EPYC 9354
RAM	24 GB
Python	v3.10.12
Pytorch	v2.0.1

**Table 2:** Results of ablation experiments.

A	B	C	*Parameters*	*GFLOPs*	*Model_Size*	*FPS*	*mAP*50	*mAP*50-90	*R*	*P*
			2,708,600	8.4	5.8 MB	56	0.932	0.69	0.886	0.941
√			1,981,424	7.8	4.3 MB	67	0.937	0.692	0.891	0.947
	√		2,244,164	7.1	4.9 MB	48	0.931	0.673	0.886	0.939
		√	2,654,200	7.9	5.7 MB	52	0.94	0.682	0.883	0.94
√	√		1,516,988	6.4	3.4 MB	71	0.935	0.686	0.895	0.948
	√	√	2,189,764	6.9	4.8 MB	50	0.929	0.685	0.892	0.945
√		√	1,933,552	7.6	4.2 MB	64	0.937	0.695	0.913	0.955
√	√	√	1,469,116	6.3	3.3 MB	77	0.942	0.682	0.898	0.953

**Table 3:** Results of ablation experiments for the detection of residues in tilapia fillets.

Model	Class	*AP*50	*AP*50-90	*R*	*P*
YOLOv10n	bone	0.917	0.608	0.896	0.942
	blood	0.994	0.764	0.981	0.987
	scale	0.927	0.634	0.859	0.889
	viscera	0.906	0.755	0.865	0.932
YOLOv10n + A	bone	0.942	0.634	0.858	0.918
	blood	0.993	0.75	0.954	1
	scale	0.914	0.619	0.854	0.948
	viscera	0.903	0.77	0.866	0.941
YOLOv10n + B	bone	0.955	0.619	0.91	0.951
	blood	0.993	0.756	0.961	1
	scale	0.899	0.606	0.786	0.906
	viscera	0.881	0.739	0.85	0.94
YOLOv10n + C	bone	0.959	0.615	0.849	0.951
	blood	0.995	0.748	1	0.968
	scale	0.921	0.623	0.845	0.888
	viscera	0.896	0.752	0.85	0.913
YOLOv10n + A + B	bone	0.95	0.65	0.887	0.931
	blood	0.993	0.773	1	0.982
	scale	0.924	0.599	0.874	0.895
	viscera	0.903	0.739	0.872	0.97
YOLOv10n + B + C	bone	0.934	0.63	0.896	0.945
	blood	0.991	0.755	0.964	1
	scale	0.924	0.629	0.885	0.938
	viscera	0.889	0.75	0.878	0.919
YOLOv10n + A + C	bone	0.942	0.632	0.944	0.935
	blood	0.993	0.77	0.987	0.999
	scale	0.941	0.628	0.903	0.94
	viscera	0.89	0.758	0.87	0.985
YOLOv10n + A + B + C	bone	0.947	0.629	0.899	0.959
	blood	0.995	0.761	0.981	1
	scale	0.928	0.625	0.893	0.916
	viscera	0.908	0.755	0.878	0.962

**Table 4:** Results of different models using dual-head GC lightweighting.

Model	*Parameters*	*GFLOPs*	*Model_Size*	*FPS*	*mAP*50	*mAP*50-90	*R*	*P*
YOLOv10s	8,069,448	24.8	16.6 MB	43	0.937	0.697	0.91	0.956
YOLOv10s + dual head GC	4,858,200	17.6	10.1 MB	53	0.943	0.706	0.918	0.959
YOLOv10l	31,205,864	144.6	63 MB	21	0.944	0.707	0.911	0.949
YOLOv10l + dual head GC	15,644,592	77.3	31.9 MB	32	0.944	0.709	0.911	0.953
YOLOv8n	3,011,628	8.2	6.3 MB	62	0.93	0.671	0.875	0.936
YOLOv8n + dual head GC	1,484,980	5.9	3.3 MB	74	0.933	0.678	0.885	0.938
YOLOv8s	11,137,148	28.7	22.6 MB	40	0.937	0.695	0.902	0.949
YOLOv8s + dual head GC	5,423,056	19.6	11.2 MB	50	0.94	0.692	0.914	0.949

**Table 5:** Detection performance of the double-headed GC-YOLOv10n compared to popular object detection algorithms.

Model	*Parameters*	*GFLOPs*	*Model_size*	*FPS*	*mAP*50	*mAP*50-90	*R*	*P*
YOLOv5n	2,509,228	7.2	5.3 MB	58	0.934	0.689	0.901	0.937
YOLOv5m	25,067,436	64.4	50.5 MB	35	0.944	0.679	0.905	0.941
Swin-Transformer	51,323,782	132.2	103.2 MB	24	0.933	0.684	0.899	0.942
RT-DERT	32,814,296	108	66.2 MB	30	0.887	0.627	0.837	0.868
ShuffleNetV2	1,373,596	4.8	3.0 MB	81	0.924	0.642	0.886	0.927
MobileNetV3	2,354,922	5.4	5.0 MB	72	0.926	0.651	0.879	0.935
ours	1,469,116	6.3	3.3 MB	77	0.942	0.682	0.898	0.953

## Data Availability

The original contributions presented in the study are included in the article, further inquiries can be directed to the corresponding author.
